# GlycoPOST realizes FAIR principles for glycomics mass spectrometry data

**DOI:** 10.1093/nar/gkaa1012

**Published:** 2020-11-11

**Authors:** Yu Watanabe, Kiyoko F Aoki-Kinoshita, Yasushi Ishihama, Shujiro Okuda

**Affiliations:** Division of Bioinformatics, Niigata University Graduate School of Medical and Dental Sciences, 1–757 Asahimachi-dori, Chuo-ku, Niigata 951–8510, Japan; Faculty of Science and Engineering, Soka University, 1–236 Tangi-machi, Hachioji, Tokyo 192-8577, Japan; Department of Molecular and Cellular BioAnalysis, Graduate School of Pharmaceutical Sciences, Kyoto University, Sakyo-ku, Kyoto 606-8501, Japan; Department of Proteomics and Drug Discovery, Graduate School of Pharmaceutical Sciences, Kyoto University, Sakyo-ku, Kyoto 606-8501, Japan; Division of Bioinformatics, Niigata University Graduate School of Medical and Dental Sciences, 1–757 Asahimachi-dori, Chuo-ku, Niigata 951–8510, Japan

## Abstract

For the reproducibility and sustainability of scientific research, FAIRness (Findable, Accessible, Interoperable and Re-usable), with respect to the release of raw data obtained by researchers, is one of the most important principles underpinning the future of open science. In genomics and transcriptomics, the sharing of raw data from next-generation sequencers is made possible through public repositories. In addition, in proteomics, the deposition of raw data from mass spectrometry (MS) experiments into repositories is becoming standardized. However, a standard repository for such MS data had not yet been established in glycomics. With the increasing number of glycomics MS data, therefore, we have developed GlycoPOST (https://glycopost.glycosmos.org/), a repository for raw MS data generated from glycomics experiments. In just the first year since the release of GlycoPOST, 73 projects have already been registered by researchers around the world, and the number of registered projects is continuously growing, making a significant contribution to the future FAIRness of the glycomics field. GlycoPOST is a free resource to the community and accepts (and will continue to accept in the future) raw data regardless of vendor-specific formats.

## INTRODUCTION

For reproducibility and sustainability of scientific research, the public sharing of raw data obtained by researchers is of paramount significance (1). The FAIRness (Findable, Accessible, Interoperable and Re-usable) of datasets is the most important principle that supports open science in the future (1–3). For genomics, the sharing of raw data from next generation sequencers (NGS) is implemented through public repositories (4). In addition, registration of gene expression data such as RNAseq into data repositories is becoming standardized (5,6). Furthermore, mass spectrometry (MS) has become the method of choice for the qualitative and quantitative characterization of complex protein and glycan mixtures (7–11), and thus a need for a repository for sharing such data has been recognized. For data in the field of proteomics, qualitative and quantitative mass spectrometry-based analyses are performed and reported. These studies may characterize relatively simple systems, such as protein complexes or much more complex mixtures, such as cell organelles, full cell lysates or different organs. Thus standards such as for data processing, common data formats and issuance of common accession numbers for submitting raw data to repositories are being promoted under the global activity called the ProteomeXchange (PX) consortium (12). In the proteome field, there are several repositories approved by this PX Consortium all over the world (13–16), and each of them operates its own repository according to their respective region and specific data format. As a result, all proteome MS data are accessible from the ProteomeCentral portal site managed by the PX Consortium, where over 20,000 projects are currently registered (17).

Proteomics analysis may also include the characterization of post-translational modifications (PTMs) including glycosylation. However, in most cases such PTMs are simply added in the annotations as text. With the recent development of a glycan structure repository GlyTouCan (18), this information should also be linked with glycan and glycomics data. Therefore, in the glycomics field, the Minimum Information Required for A Glycomics Experiment (MIRAGE) initiative began with the recommendation of minimum information required to be reported when publicizing glycomics experiments (19). MIRAGE standards for ‘minimum information required for a glycomics experiment’ and proposes guidelines for many of the experimental techniques used when working with glycans. The first of these guidelines was for MS experiments, where the minimum information needed to be reported was delineated including the type of instrument used, its parameters, peak lists with characterized structures and raw data. This guideline helped standardize the metadata required for the registration of MS data in a repository. UniCarb-DR was recently announced as a repository for characterized glycans by MS, storing peak list information, and GlycoPOST was mentioned as the raw data repository (20). In this manuscript, we describe the details on the usage of GlycoPOST.

We believe that there is an urgent need for an official repository for glycomics MS raw data, so we have developed and since operated a repository called GlycoPOST. GlycoPOST has been made available for over a year so far, and through our efforts to approach the glycomics community, 50 users have registered, >70 projects have been created, and over 2000 files have now been deposited in GlycoPOST, totalling 700 GB of data. As the use of MS in the glycoscience field is expected to grow further in the future, the number of projects registered with GlycoPOST is very likely to increase.

## DATABASE DESCRIPTION

**Figure 1.**
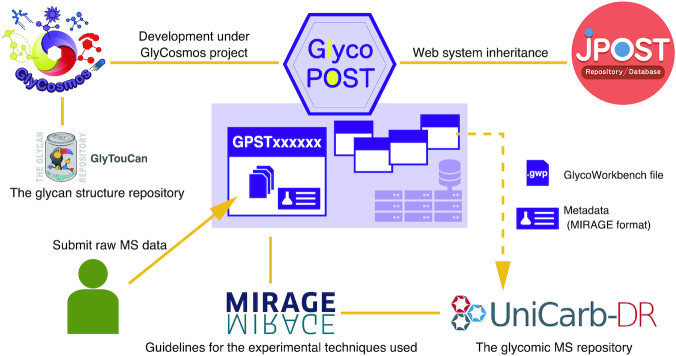
Schematic representation of the GlycoPOST environment. GlycoPOST has been developed under the GlyCosmos project, and the GlycoPOST system was adapted from the repository system of the jPOST project. The metadata to be registered follows the MIRAGE guidelines and the Excel format for the metadata input used by UniCarb-DR is also importable.

GlycoPOST accepts MS data from glycomics experiments and issues an accession number to provide traceability for reuse and reanalysis of the data. This system is an adaptation of the jPOST repository system (14), which has already proven to be a stable MS data repository for proteomics. Basically, the technology implemented in the jPOST repository has been implemented in GlycoPOST as well, and it inherits the usability of the jPOST repository. In addition, the GlycoPOST system has been designed to make it easy to input various metadata such as experimental conditions and instrument settings specific to glycomics. Metadata such as experimental conditions are set to comply with the MIRAGE guidelines, and thus we can claim that GlycoPOST contributes to standardization in the glycomics field (Figure 1). As illustrated in this figure, GlycoPOST is a part of the GlyCosmos portal (21), which also includes UniCarb-DR and GlyTouCan (18) as fellow repository systems. GlyTouCan is the international glycan structure repository, and it assigns accession numbers to individual glycans. UniCarb-DR is a repository of peak lists, and the raw data is registered in GlycoPOST. Due to this relationship between UniCarb-DR and GlycoPOST, we have implemented a combined user registration system that handles user information for both repositories.

### MIRAGE guidelines

MIRAGE (Minimum Information Required for A Glycomics Experiment) is a set of guidelines established by the MIRAGE committee to specify the minimum information required for reporting glycan-related experiments, such as sample preparation (doi:10.3762/mirage.1), mass spectrometry (doi:10.3762/mirage.2), glycan arrays (doi:10.3762/mirage.3), and liquid chromatography (doi:10.3762/mirage.4) (19). MIRAGE is supported by the Beilstein Institute in Germany, and the MIRAGE committee is made up of renowned glycomics scientists and glyco-informaticians from around the world.

GlycoPOST has adopted the guidelines for the portion of MIRAGE that is relevant to mass spectrometry experiments for glycomics. To make it easier for users to enter and manage metadata, the input section for metadata has been divided into the following five sections, ‘Sample preparation’, ‘General features’, ‘Ion sources’, ‘Ion transfer optics’, and ‘Spectrum and peak list generation and annotation’, each of which can be registered in GlycoPOST as a reusable ‘preset’. As long as the experimental conditions are the same, the user can use a previously created preset as is, or they can change some parts of it and create another preset. Note that the content of each of the following presets are all based on the current version of the MIRAGE guidelines and are subject to change based on any updates to these guidelines. The latest version of the details of this information is available at https://glycopost.glycosmos.org/help#mirage.

### Preset 1: Sample preparation

The sample preparation section is designed to include all aspects of sample generation, purification and modifications of the biological and/or synthetic material analyzed. Users input biologically derived material and/or chemically derived material as sample origin, and enzymatic and/or chemical treatments as sample processing for isolation. In addition, enzymatic and/or chemical modifications, and purification steps are needed to be registered.

### Preset 2: General features

In this preset, global descriptions such as the used instrumentation, any particular customizations, and general instrument control parameters such as instrument control software. This includes the software name and version information.

### Preset 3: Ion sources

This preset is used for summarizing all the parameters for ion generation including controls of in-source fragmentation, the degree of fragmentation, as well as other more common parameters such as capillary voltage or laser intensity settings.

### Preset 4: Ion transfer optics

This preset requires instrumental details related to the processes after ions are generated such as transport, gas phase reactions and detection of ions.

### Preset 5: Spectrum and peak list generation and annotation

The software used to generate peak list files from mass spectrometry raw data files and software and/or databases used to annotate each spectrum are needed to be input. This category is optional because it is often not possible to obtain this data.

In addition, UniCarb-DR (https://unicarb-dr.glycosmos.org/) provides a web tool that allows users to enter MIRAGE-related information for their experiments, which produces an Excel file formatted in a specific format with the required information (20). GlycoPOST has a function to import the data from this Excel spreadsheet and automatically create presets. Conversely, users can also export the preset data from GlycoPOST and download an Excel spreadsheet in the same format.

Thus, we made efforts to ensure compatibility with other glycomics data repositories. Furthermore, by adopting the MIRAGE guidelines, we can ensure the quality of the metadata registered in GlycoPOST and that it is compatible with databases in related fields.

### Project creation and file upload

**Figure 2.**
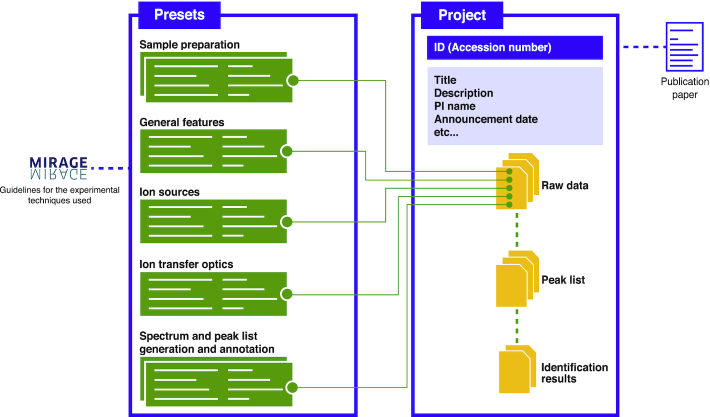
Relationships between data files and metadata. Presets that follow the MIRAGE guidelines are registered in GlycoPOST, and each preset is linked to raw data files obtained from mass spectrometry. In addition to the raw data, a project is created that contains the peak list and result files, and an accession number is issued for that project.

In general, users would register their data as a single project, which receives a unique accession number. Each project can contain one or more raw data and must be linked with metadata as defined by the presets described previously. Each project is required to be linked with at least Presets 1–4 for describing samples, experiments, and instruments, and once registered, and one accession number will be issued. After a project is generated, any metadata for sample preparation, general features, ion sources, ion transfer, spectrum and peak list registered as a preset will be linked to the MS raw data files to be registered (Figure 2). The same metadata information can be linked to all files at once by drag-and-drop of the raw data files into the browser with presets selected beforehand, greatly reducing the registration operation for users with many files to register.

After linking the metadata profiles as presets with the deposited data files, the user can upload the files to the repository. GlycoPOST utilizes the PRESTO system (https://prestotools.github.io/) for uploading data. The upload process of this system splits the file into smaller pieces, called ‘chunks’, which are then uploaded to the repository in parallel. In the process of data transmission over the Internet, it is known that the longer the distance of the data communication route, the greater the delay (the delay before the data actually starts to be transferred). This often results in very slow data transfer rates between physically distant locations. This delay problem can be remedied by uploading small chunks in parallel.

This data transfer system is already implemented in the jPOST repository, and it has shown to have a positive correlation between file size and transfer time, with an average transfer rate of about 5MB/s, which is fast enough to take only about four minutes to upload a 1GB file (14). Thus, it has been found that the file transfer speed is, in most cases, independent of the distance from where the user deposits the data in this system.

### Data publication and download

When the deposited dataset is determined to be valid against the MIRAGE guideline criteria, the users can lock and exit the submission process, at which time a GlycoPOST identifier is generated as an accession number. The submitted data and metadata are automatically checked by our system and if the dataset is determined to be incomplete, an accession number will not be assigned and the dataset cannot be announced. At this stage of a ‘project’ submission, datasets submitted to the repository are in ‘embargo’, meaning it is set as private, and it will be automatically published on a ‘publication date’ set by the users themselves. During this embargo period, users will be issued a dedicated URL and password that will allow anyone with this information, such as journal editors and peer reviewers, to access the project. Users can also revise a temporarily locked project in response to reviewers’ comments and revised data will be assigned a revision number.

Datasets of published projects can be downloaded without restrictions. Users can also search for keywords found in any of the fields registered under presets or projects.

### System implementation

The web application for GlycoPOST was built using the React framework (https://reactjs.org/), and the proprietary PRESTO system is used for file uploads. This eliminates the need for FTP and external software for file uploads, allowing the entire process from project creation to file uploads to be completed within a single web browser, contributing to an improved user experience.

## DISCUSSION

**Figure 3.**
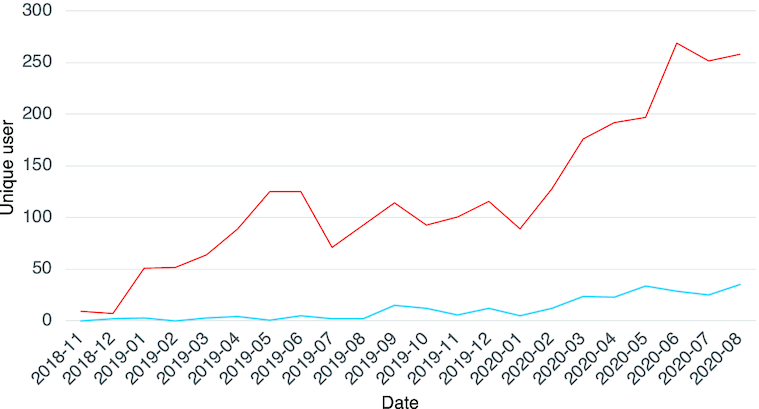
History of user access. Red line indicates the number of unique users accessed to the top page of GlycoPOST and blue line indicates the number of unique users downloaded the data stored in GlycoPOST.

The alpha version of GlycoPOST was launched in December 2018, with the beta version released in April 2019, and its official release in March 2020. During this time, it has been used by many users, with over 70 projects registered, of which >20 are in the public domain. Over half of the registered projects are based on ESI-MS/MS analysis, but others include ESI-MS, MALDI-MS and MALDI-MS/MS, which are the instrumentation listed by the MIRAGE guidelines. However, other technologies can be selected under ‘Not specified’ for the time being. As more data using these other technologies are deposited, they will be added to the predefined list. The numbers of datasets deposited using positive and negative mode were about half and half. Regarding glycan labeling and derivatization, currently there is no controlled vocabulary, so users have entered free text to describe this under the sample processing section. Those who used glycan labeling will have specified this information, but unlabeled glycans would not be mentioned. All the required metadata have been specified by all users since the official release of GlycoPOST in April, 2020. The number of accesses and downloads have increased in general, as shown in Figure 3. Although the server itself is located in Japan, it has attracted a lot of attention as it is used by researchers worldwide not only Asia but also North America and Europe. We assume that this is due in large part to the need that it fulfills and its usability.

All metadata submitted to this repository, especially the experimental procedures described in the current five presets, are not currently represented as any ontology or controlled vocabulary. This is due to the fact that although the MIRAGE guidelines exist, no repository could fully accommodate the guidelines until now. Because many other glycomics-related databases already use ontologies to represent glycan-related information (18,21), by increasing the use of ontologies in GlycoPOST in the future and expressing them in a unified framework such as the Resource Description Framework (RDF) data model, it should become possible to integrate the data in GlycoPOST with other glycan-related databases (2,3,22). Moreover, MIRAGE has yet to publish a glycoproteomics guideline, but there are plans to make one available soon in collaboration with the HUPO Proteomics Standards Initiative (PSI) (23). As soon as these guidelines are complete and announced, we plan on implementing functionality to accept the relevant metadata in GlycoPOST to be able to accept glycoproteomics data. This will prepare us to apply to the ProteomeXchange as a fellow member. The MIRAGE guidelines will delineate the metadata for glycan structure information, which will be linked with GlyTouCan and other related glycan resources; this is currently lacking in proteomics repositories.

Currently, UniCarb-DR and GlycoPOST are independent systems except for user information. In the near future, the data between these repositories will be shared, so that the raw data registered in GlycoPOST can be mapped to the peak lists and glycans registered in UniCarb-DR, and vice-versa. Moreover, these glycan data will be linked to GlyTouCan accession numbers. As a result, the raw data in GlycoPOST can be visualized with the spectra of the glycan fragments registered in UniCarb-DR. Moreover, this workflow can be made more seamless by having users first register the raw data, peak lists and glycan data in GlycoPOST, which then automatically registers the glycan data into UniCarb-DR to take advantage of the latter's connection with GlyTouCan. Then, by integrating all this information under a common framework, glycans can be searched throughout UniCarb-DR and GlycoPOST in the future. This will make re-analysis of the glycomics MS data in GlycoPOST easier, by allowing users to search for raw data containing a particular glycan.

Making data findable, accessible, interoperable and re-usable will not only enhance its value as a public asset, but also contribute to the many studies that will help us create new value. In the field of glycomics, this concept of FAIRness is an important common philosophy that needs to be realized in the same way as in genomics and proteomics. This will allow for re-analysis of the data as detection algorithms and technologies improve, especially considering that de novo analysis is currently extremely difficult. Moreover, by working with journals to require the submission of raw data to a repository, the data will be more accessible for other users, where metadata will aid in searching for the most appropriate datasets and the guidelines ensure that the data is accessible in a standard format. We believe that GlycoPOST will be a major contributor to these public roles.
